# Physiological and transcriptome analysis of *Poa pratensis* var. *anceps* cv. Qinghai in response to cold stress

**DOI:** 10.1186/s12870-020-02559-1

**Published:** 2020-07-31

**Authors:** Wenke Dong, Xiang Ma, Hanyu Jiang, Chunxu Zhao, Huiling Ma

**Affiliations:** 1 Key Laboratory of Grassland Ecosystem of Ministry of Education, College of Grassland Science, Gansu Agricultural University, Lanzhou, 730070 China; 2 Key Laboratory of Superior Forage Germplasm in the Qinghai-Tibetan Plateau, Qinghai Academy of Animal Science and Veterinary Medicine, Xining, 810016 China; 3 Department of Physic, Nanjing Normal University, Nanjing, 210097 China

**Keywords:** Cold stress, *Poa pratensis* var. *anceps* cv. Qinghai, Phenotypic and physiological changes, Transcriptome, Differentially expressed genes

## Abstract

**Background:**

Low temperature limits the growth and development and geographical distribution of plants. *Poa pratensis* is a cool-season turfgrass mainly grown in urban areas. However, low winter temperature or cold events in spring and autumn may cause *P.pratensis* mortality, affecting the appearance of lawns. *P.pratensis* var. *anceps* cv. Qinghai (PQ) is widely distributed in the Qinghai-Tibet Plateau above 3000 m. PQ has greater cold tolerance than the commercially cultivated *P.pratensis* varieties. However, existing studies on the response mechanism of PQ to low temperatures have mainly focused on physiological and biochemical perspectives, while changes in the PQ transcriptome during the response to cold stress have not been reported.

**Results:**

To investigate the molecular mechanism of the PQ cold response and identify genes to improve the low-temperature tolerance of *P.pratensis*, we analyzed and compared the transcriptomes of PQ and the cold-sensitive *P.pratensis* cv. ‘Baron’ (PB) under cold stress using RNA sequencing. We identified 5996 and 3285 differentially expressed genes (DEGs) between the treatment vs control comparison of PQ and PB, respectively, with 5612 DEGs specific to PQ. Based on the DEGs, important Kyoto Encyclopedia of Genes and Genomes (KEGG) pathways, such as “starch and sucrose metabolism”, “protein processing in endoplasmic reticulum”, “phenylalanine metabolism” and “glycolysis/gluconeogenesis” were significantly enriched in PQ, and “starch and sucrose metabolism”, “phenylpropanoid biosynthesis”, “galactose metabolism” and “glutathione metabolism” were significantly enriched in PB. In addition, the “glycolysis” and “citrate cycle (TCA cycle)” pathways were identified as involved in cold tolerance of *P.pratensis*.

**Conclusions:**

As we know, this is the first study to explore the transcriptome of *P.pratensis* var. *anceps* cv. Qinghai. Our study not noly provides important insights into the molecular mechanisms of *P.pratensis* var. *anceps* cv. Qinghai responds to cold stress, but also systematically reveals the changes of key genes and products of glycolysis and TCA cycle in response to cold stress, which is conductive to the breeding of cold-tolerance *P.pratensis* genotype.

## Background

Cold stress, as a common environmental stress factor, is one of the leading factors limiting the growth, development, and geographical distribution of plants [1]. When plants are under cold stress, they will show some symptoms such as surface lesions, water loss/desiccation, tissue breakdown, accelerated senescence/ethylene production, faster decay and death [2]. However, plants can also respond to cold stress by changing their phenotypic and physiological characteristics, such activating relative signal pathways [3], inducing antioxidant enzymes and the membrane systems [4]. The adaptation of plants to cold stress and the underlying regulatory systems comprise a comprehensive response involving diverse system regulation at the biological level, such as genetic regulation, posttranscriptional regulation, posttranslational modification, and metabolic feedback [5]. It is of great significance to study how plants respond to these biological processes by altering their genetic expressions. However, these changes may include the expression of numerous related genes. Therefore, it is difficult to reveal the complex network of the cold response system by investigating a single metabolic pathway alone.

Under cold stress, plants will produce a series of responses. These responses mainly include signaling cascade, modification of the membrane lipid composition, scavenging of reactive oxygen species (ROS), synthesis of osmoprotectants [6], and the changes in the expression level of genes related to cold stress responses. The signaling transduction pathways that regulate cold-responsive gene expression are key processes in plants responding to cold stress. Presently, the three most explored and reported pathways are abscisic acid (ABA) signaling pathway induced by ABA accumulation [7], dehydration-responsive element-binding protein 1 s (*DREB1*)/C-repeat binding factors (*CBFs*) pathway initiated by calcium signaling cascade [8], and mitogen-activated protein kinase (MAPK) cascade pathway activated by ROS [9], respectively. ABA is considered as a stress hormone, and its biosynthetic process is one of the fastest metabolic reactions in plants [10], which can regulate stomatal closure, reduce water loss and maintaine cell growth [11]. The *DREB1/CBFs* is one of the central pathways for the cold response, and regulates a subset of cold-responsive (COR) genes by binding the dehydration-responsive-element/C-repeat (*DRE/CRT*) cis-elements in the promoter regions of COR genes [12, 13]. The MAPK cascade pathway is also the most important signal pathway for regulating cold responsive genes, which mainly involves several families of TFs [14], second messengers (ROS and Ca^2+^) and some plant hormones. The ROS induces the expression of TFs through the accumulation of plant hormones, and TFs reduces the damage of ROS to plants by regulating the expression of genes related to ROS scavenger [15]. Although more and more COR genes are recognized and annotated in different plant species, different species may show different cold response mechanisms. Therefore, we need to constantly explore the regulatory pathways and genes of cold response in different species.

Transcriptome sequencing based on a high-throughput sequencing platform is able to uncover the global transcriptional activities of any species at the mononucleotide level [16]. In recent years, transcriptomics has provided a new perspective for research on the plant response to cold stress, and many studies of this kind have been reported [17–19]. Many COR genes have been identified from various species, including *Arabidopsis thaliana* [20], *Oryza sativa* [21], *Zea mays* [22], etc. In addition, the functions and metabolic responses of DEGs in several important precocious subfamilies of perennial grass under cold stress have also been identified using transcriptome techniques. These grass species include *Leymus chinensis* [23], *Lolium perenne* [24] and *Brachypodium distachyon* [25]. It is therefore of great theoretical and practical significance for cold-tolerance breeding of plants to use transcriptome techniques to study genome-wide differential expressions of genes in plants under cold stress, combined with homologous cloning to search for key genes associated with cold tolerance.

*Poa pratensis*, which is an excellent gramineous forage plant, is also a cool-season turfgrass mainly grown for turf establishment in urban areas and widely planted for construction of grasslands and ecological management. However, low temperature is the main environmental factor influencing the active growth and dormant periods of *P.pratensis*. In winter or during low-temperature events in spring and autumn, *P.pratensis* is likely to be unable to live or may become green late in the spring or senesce early in the autumn, thus influencing the appearance of the lawn [26]. At present, there are few studies on how to improve the cold tolerance of *P.pratensis*. The way to improve the cold tolerance of lawn mainly depends on the field management, such as reasonable fertilization [27]. *P.pratensis* var. *anceps* cv. Qinghai (PQ), as a variant of *P.pratensis* that grows widely in the natural grasslands of the Qinghai-Tibet Plateau, China at altitudes exceeding 3000 m. With its strong root system, ease in forming turf, and strong soil-reinforcement ability, PQ plays an important role in ecological management and water and soil conservation in the high and cold areas of the Qinghai-Tibet Plateau. Moreover, PQ is more cold tolerance than the commercially cultivated varieties of *P.pratensis* and is able to safely maintain a dormant state at temperatures as low as − 35 °C [28]. To the best of our knowledge, existing studies on the response mechanism of PQ to low temperatures have mainly focused on physiological and biochemical perspectives, while changes in the PQ transcriptome during the response to cold stress have not been reported.

In previous reports, Zhang et al. analyzed the transcriptome of *P.pratensis* cv. ‘Merit’ under cold stress, and identified some DEGs related to cold stress, such as cold-induced proteins, antioxidant enzymes, and osmoregulation proteins [29]. However, the metabolic pathways involved in these DEGs have not been analyzed in depth. In this study, we compared the phenotypic and physiological differences between the cold-tolerance PQ and the cold-sensitive *P.pratensis* cv. ‘Baron’ (PB). Then, the different molecular responses of the two *P.pratensis* genotypes to cold stress were analyzed using transcriptomic methods, and the related metabolic pathways were studied in depth. The research results provide a basis for gaining more specific knowledge of the molecular mechanism of the PQ adaptation to cold stress and for searching for genes with potential for improving the low-temperature tolerance of *P.pratensis*.

## Results

### Phenotypic and physiological responses to cold stress

**Fig. 1:**
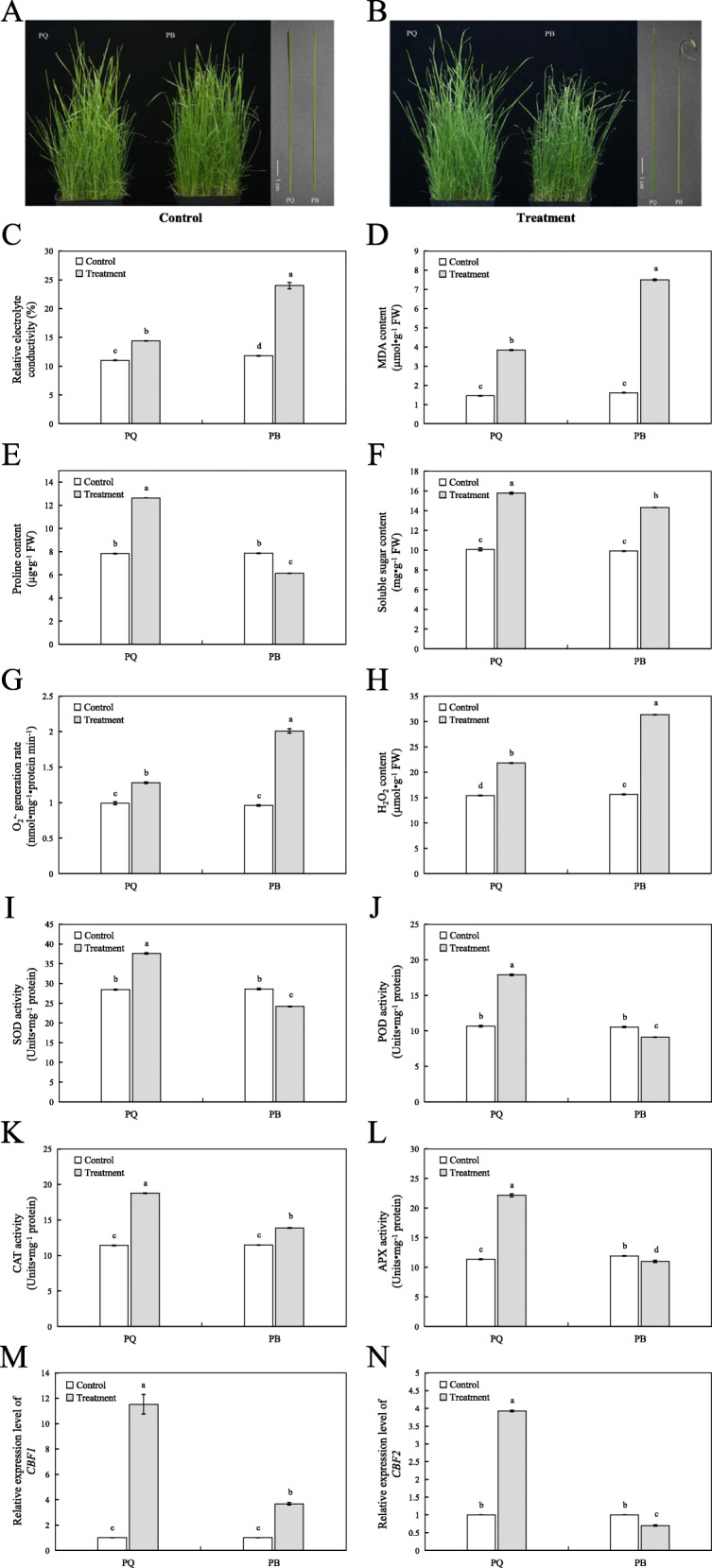
Phenotypic and physiological responses of two *P.pratensis* cultivars under control and cold stress conditions. Phenotypic change (**a** and **b**), the REC (**c**), MDA (**d**), Pro (**e**), SS content (**f**), O_2_^·-^ generation rate (**g**), H_2_O_2_ content (**h**), SOD (**i**), POD (**j**), CAT (**k**) and APX (**l**) activity, *CBF1* (**m**) and *CBF2* (**n**) expression level were measured. All data are presented as means±SE from three independent experimental replicates. Different letters indicate significantly different at *p* < 0.05

Under cold stress, the phenotypic and physiological changes of the two genotypes were significant (Fig. 1). Under the control conditions, the growth of the two genotypes was good, and the leaves did not wither or die (Fig. 1a). After 72 h of cold treatment and 24 h of recovery, the leaf morphology of PQ did not change significantly, but the leaf tips of PB had dried and withered (Fig. 1b). Relative electrolyte conductivity (REC) and malondialdehyde (MDA) are commonly used to measure membrane damage and cell stability. Under cold stress, the membrance system and the balance of ROS metabolism in plant cells are destroyed [30]. Therefore, the REC and MDA contents of PQ and PB increased significantly under cold stress. However, the increase in PQ was lesser than that in PB. The increase rate of REC was 30.76% vs 98.30%, and that of MDA was 164.83% vs 357.67% (Fig. 1c and d). Free proline (Pro) and soluble sugar (SS) help maintain cell membrane stability [31]. Compared with the control conditions, the Pro content of PQ increased by 61.30%, while it decreased by 24.94% in PB (Fig. 1e). The SS content of the two genotypes increased significantly under cold stress. In contrast to the control conditions, the SS content increased by 53.20% in PQ and by 45.14% in PB (Fig. 1f). Under cold stress, the superoxide radical (O_2_^·-^) generation rate and hydrogen peroxide (H_2_O_2_) content of the two genotypes were significantly increased compared with that under the control conditions (Fig. 1g and h). However, the O_2_^·-^ generation rate and H_2_O_2_ content of PQ were both lower than those of PB (Fig. 1g and h). Moreover, we also determined the antioxidant enzyme activities in the leaves of the two genotypes. The results showed that the superoxide dismutase (SOD), peroxidase (POD), hydroperoxidase (CAT) and ascorbate peroxidase (APX) activities of PQ were significantly improved compared with those of the control conditions (Fig. 1i, j, k and l). However, in PB, the activities of SOD, POD and APX were significantly lower than those in the control conditions, with the exception of CAT activity (Fig. 1i, j, k and l). The C-repeat binding factor (*CBF*)/dehydration responsive element binding factor (*DREB*) is one of the most effective pathways in the cold response of plants [32]. Similar to the above physiological changes, the expression levels of *CBF1* and *CBF2* in PQ were significantly higher than those in PB under cold stress (Fig. 1m and n). These results indicated that PQ is more tolerance to cold than PB.

### RNA sequencing (RNA-seq) and transcriptome assembly

To analyze the transcriptome and gene expression profiles of the two genotypes under control and cold stress conditions, 12 cDNA samples were extracted from the leaves of the two genotypes and sequenced using the Illumina HiSeq 2500 platform. Each sample was sequenced with more than 42 million raw reads, more than 41 million clean reads, and more than 6.20 G of clean bases (Additional file 1: Table S1). Moreover, the base error rate was low at 0.02%, the Q20 percentage was over 97.00%, and the Q30 percentage was over 93.00% (Additional file 1: Table S1). The GC content was between 55.49 and 56.87%. A total of 573,862,822 raw reads were obtained from the 12 samples (Additional file 1: Table S1). After removing the low quality reads and adaptor sequences, 567,006,774 clean reads were obtained (Additional file 1: Table S1). The capacity of the clean bases was 84.45 G (Additional file 1: Table S1). All clean data were then de novo assembled by Trinity software, and the assembly results were evaluated. A total of 944,479 transcripts were obtained from 12 samples. The average length of the transcripts was 454.62 bp, and the N50 length was 502 bp (Additional file 2: Table S2). Moreover, 458,419 unigenes were obtained with an average length of 326.19 bp, and the N50 length was 336 bp (Additional file 2: Table S2).

### Gene annotation and functional classification

To analyze and predict the function of the unigenes, we performed functional annotation of all the unigenes obtained by transcriptome assembly through BLAST and six public databases. Of the 458,419 unigenes, 183,701 (40.07%) were significantly matched in at least one of the six databases. Among them, 174,005 (37.96%), 98,674 (21.52%), 80,568 (17.58%), 14,768 (3.22%), 130,023 (28.36%) and 61,759 (13.47%) unigenes were found in the Non-Redundant Protein Sequence Database (NR), Swiss-prot, Pfam, Clusters of Orthologous Groups of proteins (COG), Gene Ontology (GO) and Kyoto Encyclopedia of Genes and Genomes (KEGG) databases, respectively (Additional file 3: Table S3). The NR database produced the most matches. The other species that were compared with *P.pratensis* were *Aegilops tauschii* (32.59%), *Brachypodium distachyon* (20.49%), *Hordeum vulgare* (11.07%) and *Triticum urartu* (9.34%) (Additional file 4: Figure S1A).

GO analyses were used to classify the functions of predicted *P.pratensis* unigenes. A total of 130,023 unigenes were successfully annotated and classified into three main categories, including biological processes, cellular components, and molecular functions. Cellular (56,852), metabolic (53,563), single-organism (32,677), cell (53,360), cell part (52,984), membrane (44,360), binding (75,473), catalytic activity (68,189) and transporter activity (7212) were the most dominant terms in the three categories (Additional file 4: Figure S1B).

To further predict unigene function and assess the integrity of the transcriptome, we searched for all unigenes in the COG database. Based on the COG database, 14,768 unigenes were classified into 25 functional categories. The “translation, ribosomal structure and biogenesis” (938) cluster represented the largest group, followed by “general function prediction only” (753), “posttranslational modification, protein turnover, chaperones” (730), “signal transduction mechanisms” (648), “replication, recombination and repair” (497), “carbohydrate transport and metabolism” (447), “function unknown” (421) and “energy production and conversion” (408) (Additional file 4: Figure S1C).

### Identification of differentially expressed genes (DEGs) in response to cold stress

**Fig. 2:**
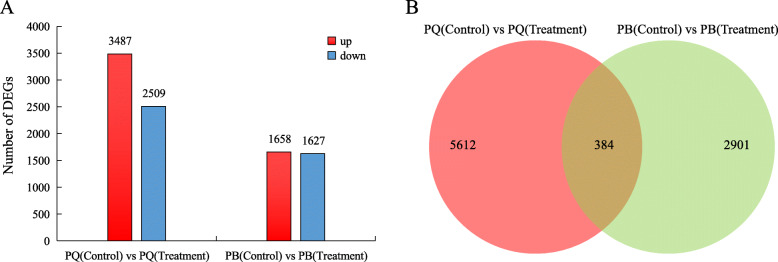
Comparison of the number of DEGs. (**a**) The number of DEGs in PQ and PB under cold stress; (**b**) The number of DEGs common between, and specific to, PQ and PB under cold stress

The expression level of each unigene was analyzed by calculating the transcripts per kilobase of exon model per million mapped reads (TPM). Compared with the control conditions, PQ and PB showed significant changes in gene expression under cold stress, but the number of changed genes was different. In PQ, 5996 DEGs were identified, including 3487 up-regulated and 2509 down-regulated DEGs (Fig. 2a). In PB, 1658 and 1627 DEGs were up-regulated and down-regulated, respectively (Fig. 2a). The number of DEGs obtained by comparing the two genotypes was counted, and their common DEGs and unique DEGs are shown in the venn diagram (Fig. 2b). The results showed that 5612 DEGs were specific to PQ, 2901 were specific to PB and 384 DEGs shared the same patterns between the two genotypes (Fig. 2b).

### Gene ontology (GO) enrichment analysis of DEGs

**Fig. 3:**
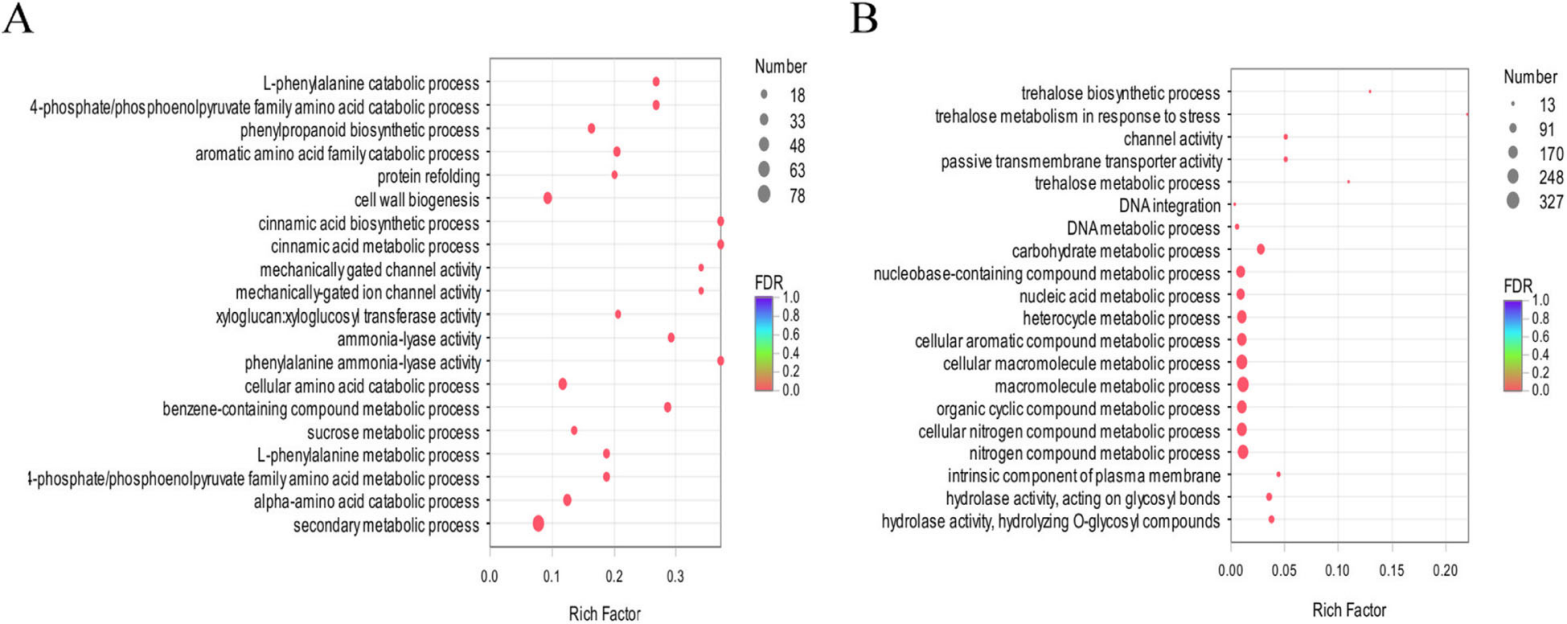
The 20 most enriched GO terms among the DEGs identified in PQ (**a**) and PB (**b**)

GO enrichment analysis was performed on DEGs of the two genotypes. The results showed that the 5996 DEGs were categorized into 1151 GO terms in PQ, including three classifications: “biological processes” (671), “cellular components” (138) and “molecular functions” (342) (Additional file 5: Figure S2). Based on the corrected *P*-values, we selected the 30 most enriched GO terms, and the primary biological process terms were “metabolic process” (1629 genes), “cellular process” (1568 genes) and “single-organism process” (1041 genes) (Additional file 5: Figure S2). The primary cellular component terms were “cell” (1646 genes), “cell part” (1631 genes) and “membrane” (1341 genes). The primary molecular function terms were “catalytic activity” (2008 genes) and “binding” (1862 genes). According to the bubble chart display, the most significantly enriched GO terms were “secondary metabolic process” (GO: 0019748), “cell wall biogenesis” (GO: 0042546), “cellular amino acid catabolic process” (GO: 0009063) and “alpha-amino acid catabolic process” (GO: 1901606) (Fig. 3a).

The 3285 DEGs were categorized into 650 GO terms in PB, including three classifications: “biological processes” (360), “cellular components” (65) and “molecular functions” (225) (Additional file 5: Figure S2). Among the 30 most abundant GO terms, the primary biological process terms were “metabolic process” (810 genes), “cellular process” (713 genes) and “single-organism process” (541 genes). The primary cellular component terms were “cell” (790 genes), “cell part” (722 genes) and “membrane” (698 genes) (Additional file 5: Figure S2). The primary molecular function terms were “catalytic activity” (1081 genes) and “binding” (904 genes). In terms of the biological process, cellular component, and molecular function categories, the most significantly enriched GO terms were “macromolecule metabolic process” (GO: 0043170), “cellular macromolecule metabolic process” (GO: 0044260) and “nitrogen compound metabolic process” (GO: 0006807) (Fig. 3b).

### KEGG pathway enrichment analysis of DEGs

**Fig. 4:**
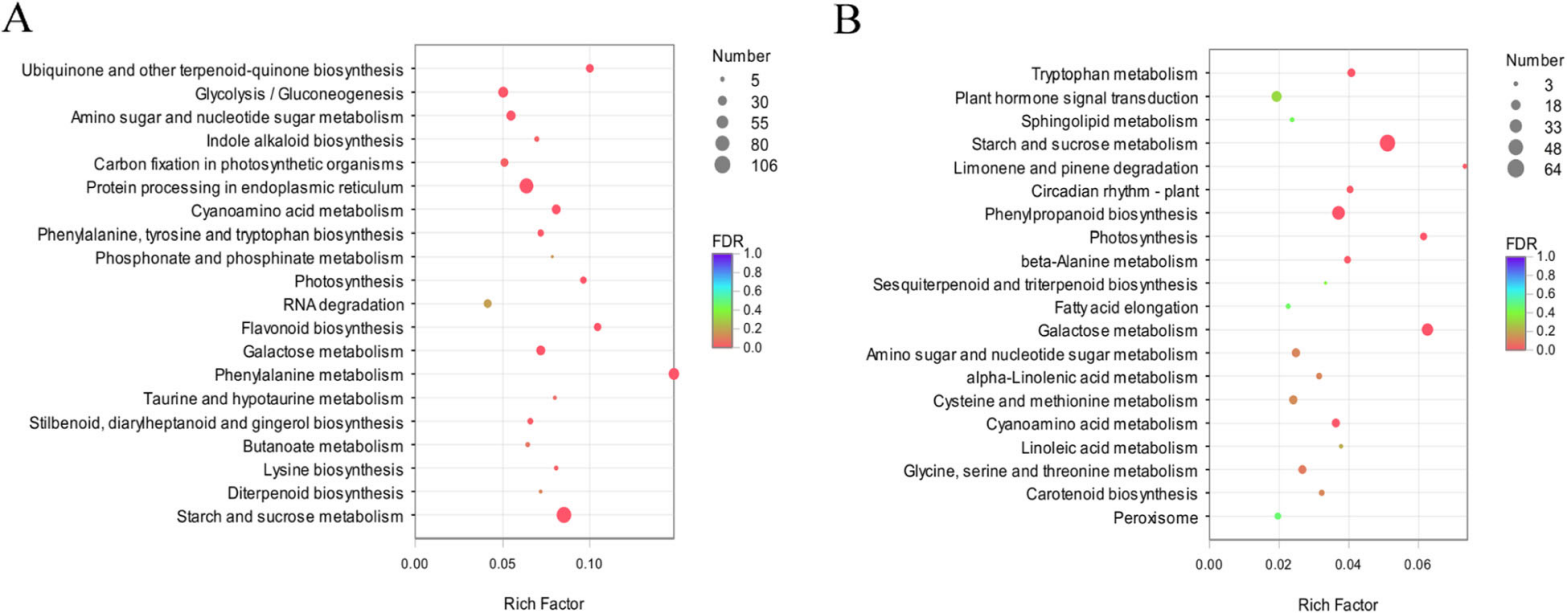
The 20 most enriched KEGG pathways among the DEGs identified in PQ (**a**) and PB (**b**)

To understand the functions of the DEGs, we mapped the DEGs to reference canonical pathways in the KEGG database. The DEGs in PQ were assigned to 119 KEGG pathways. Most of the pathways were grouped into the “Metabolism” category, such as “carbohydrate metabolism” (225 genes), “amino acid metabolism” (190 genes) and “energy metabolism” (96 genes), followed by “Genetic information processing” and “Environmental information processing” category (Additional file 6: Figure S3A). Based on the number of enriched DEGs, we listed the top 20 pathways (Fig. 4). The most significantly enriched pathways were “starch and sucrose metabolism” (ko00500), “protein processing in endoplasmic reticulum” (ko04141), “phenylalanine metabolism” (ko00360) and “glycolysis/gluconeogenesis” (ko00010) (Fig. 4a). The DEGs in PB were assigned to 109 KEGG pathways. Similarly, most of the pathway were grouped into the “Metabolism” category, such as “carbohydrate metabolism” (112 genes), “energy metabolism” (45 genes) and “metabolism of other amino acids” (31 genes) (Additional file 6: Figure S3B). Enrichment analysis showed that the significantly enriched KEGG pathway were “starch and sucrose metabolism” (ko00500), “phenylpropanoid biosynthesis” (ko00940), “galactose metabolism” (ko00052) and “glutathione metabolism” (ko00480) (Fig. 4b). The above results indicated that cold stress affects the “starch and sucrose metabolism” pathways of the two genotypes. Comparing the KEGG pathways of PQ and PB, we were most interested in pathways with higher enrichment in PQ but lower enrichment in PB, such as “glycolysis/gluconeogenesis” and “citrate cycle (TCA cycle)”. We further analyzed its relationship with the cold tolerance of *P.pratensis*.

### DEGs involved in glycolysis and citrate cycle (TCA cycle)

**Fig. 5:**
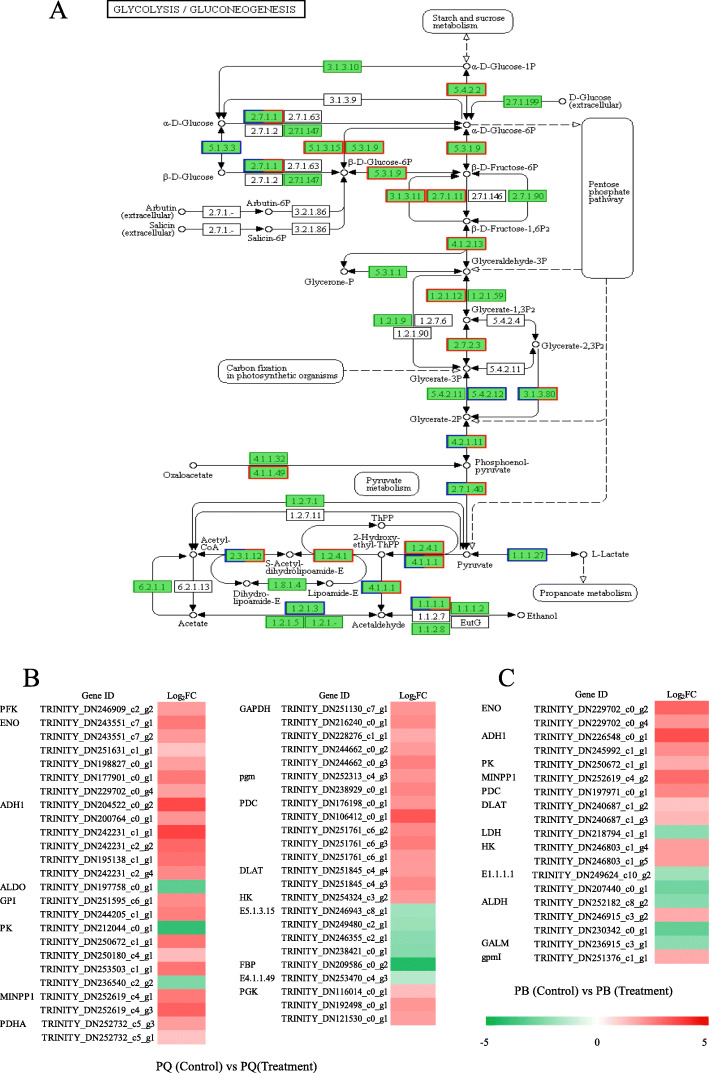
DEGs relevant to the glycolysis under cold stress. Regulatory changes in the glycolysis pathway (**a**), the heat map of the expression of DEGs related to glycolysis pathway in PQ (**b**) and PB (**c**). Red boxes indicate that DEGs were regulated in PQ under cold stress, and blue boxes indicate that DEGs were regulated in PB under cold stress. Both red and blue boxes indicate that DEGs were regulated in both genotypes

Glycolysis, as a key respiratory pathway that provides ATP, reducing agents, and metabolites for plant growth and development [33]. In this study, 49 DEGs were identified from PQ to participate in the glycolysis pathway. Forty DEGs encoding key enzymes related to glycolysis were significantly upregulated (Fig. 5b). These were phosphoglucomutase (EC 5.4.2.2), hexokinase (EC 2.7.1.1), glucose-6-phosphate isomerase (EC 5.3.1.9), 6-phosphofructokinase (EC 2.7.1.11), glyceraldehyde 3-phosphate dehydrogenase (EC 1.2.1.12), phosphoglycerate kinase (EC 2.7.2.3), 2,3-bisphosphoglycerate 3-phosphatase (EC 3.1.3.80), enolase (EC 4.2.1.11), pyruvate kinase (EC 2.7.1.40), pyruvate dehydrogenase E2 (EC 2.3.1.12), pyruvate decarboxylase (EC 4.1.1.1), and alcohol dehydrogenase (EC 1.1.1.1) (Fig. 5a). Under cold stress, 19 DEGs in PB participated in this pathway (Fig. 5c). Thirteen DEGs encoding proteins involved in glycolysis were significantly up-regulated. These DEGs included hexokinase, 2,3-bisphosphoglycerate-independent phosphoglycerate mutase (EC 5.4.2.12), 2,3-bisphosphoglycerate 3-phosphatase, enolase, pyruvate kinase, pyruvate dehydrogenase E2, pyruvate decarboxylase, aldehyde dehydrogenase (EC 1.2.1.3), and alcohol dehydrogenase (Fig. 5a).

**Fig. 6:**
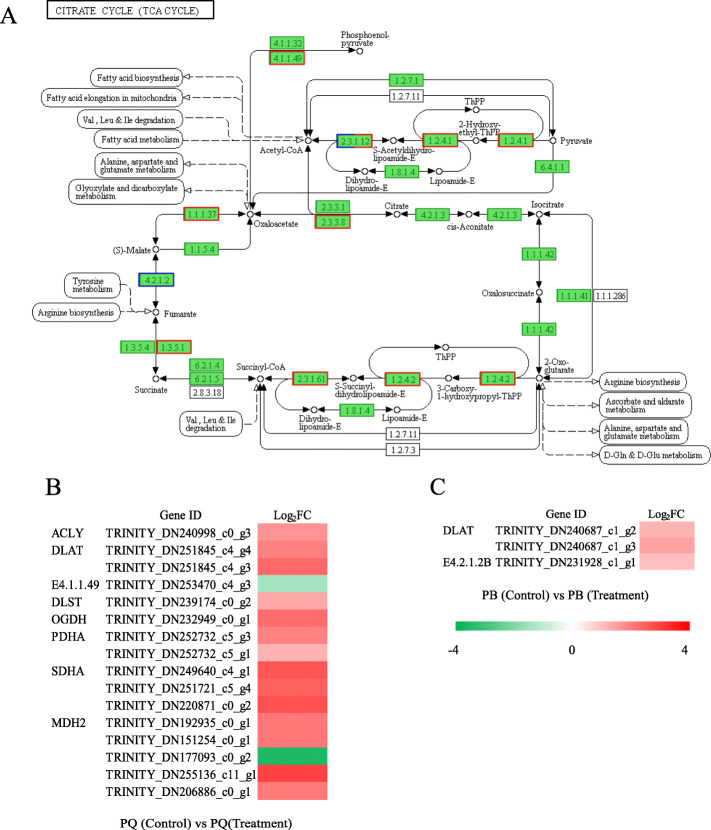
DEGs relevant to the citrate cycle (TCA cycle) pathway under cold stress. Regulatory changes in the citrate cycle (TCA cycle) pathway (**a**), the heat map of the expression of DEGs related to citrate cycle (TCA cycle) pathway in PQ (**b**) and PB (**c**). Red boxes indicate that DEGs were regulated in PQ under cold stress, and blue boxes indicate that DEGs were regulated in PB under cold stress. Both red and blue boxes indicate that DEGs were regulated in both genotypes

The citrate cycle (TCA cycle) is an important carbon metabolic pathway that occurs in mitochondria of higher plants. It is the main method of aerobic decomposition of glucose during respiration, which gradually oxidizes and decomposes the glycolytic product pyruvate into CO_2_ and H_2_O_2_, and generates NADH, FADH_2_ and ATP to support stress tolerance. In our study, 16 DEGs (14 up- and 2 down-regulated) (Fig. 6b), primarily including some key enzymes, such as pyruvate dehydrogenase E2 (EC 2.3.1.12), pyruvate dehydrogenase E1 (EC 1.2.4.1), ATP citrate (pro-S)-lyase (EC 2.3.3.8), malate dehydrogenase (EC 1.1.1.37), succinate dehydrogenase (EC 1.3.5.1), 2-oxoglutarate dehydrogenase E2 (EC 2.3.1.61), 2-oxoglutarate dehydrogenase E1 (EC 1.2.4.2), and phosphoenolpyruvate carboxykinase (EC 4.1.1.49), were identified from PQ as participating in the TCA cycle pathway (Fig. 6a). However, only 3 DEGs (3 up-regulated) were identified from PB to participate in the “TCA cycle” pathway (Fig. 6c). These were pyruvate dehydrogenase E2 (EC 2.3.1.12) and fumarate hydratase (EC 4.2.1.2) (Fig. 6a).

### Effect of carbohydrate, glycolysis and TCA cycle products content and related enzyme activities of two *P.pratensis* cultivars under cold stress

**Fig. 7:**
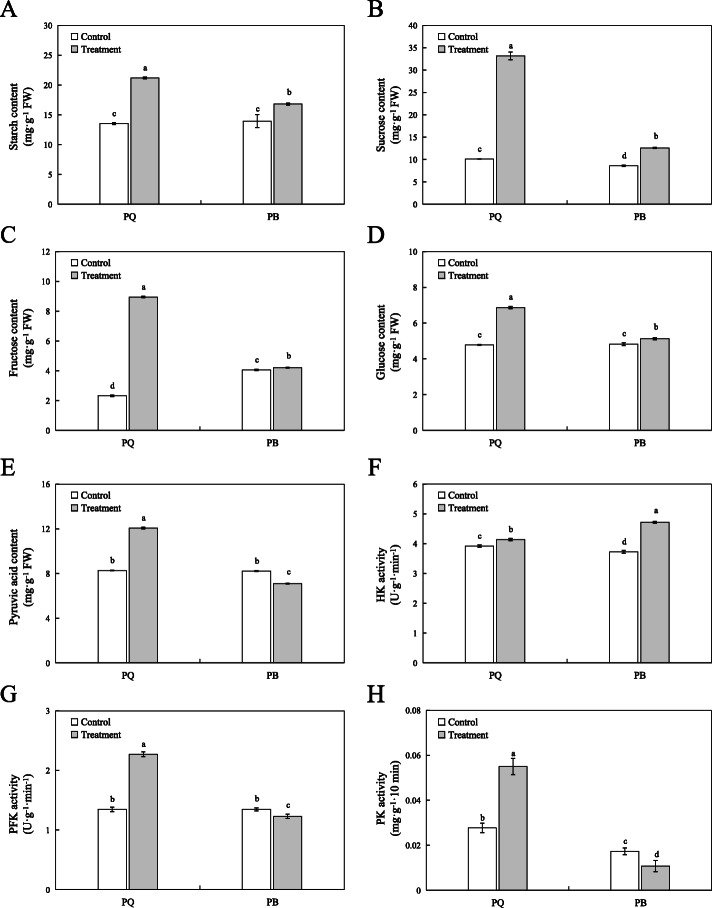
Effect of the carbohydrate and glycolysis products content and related enzyme activities of two *P.pratensis* cultivars under cold stress. The starch (**a**), sucrose (**b**), fructose (**c**), glucose (**d**), pyruvic acid content (**e**), HK (**f**), PFK (**g**), and PK (**h**) activity were measured. All data are presented as means±SE from three independent experimental replicates. Different letters indicate significantly different at *p* < 0.05

The starch, sucrose, fructose and glucose contents of PQ and PB increased significantly under cold stress, but the increase in PQ was higher than that in PB (the increase rate was 60.95% vs 19.56, 229.31% vs 49.23, 68.61% vs 4.73 and 44.75% vs 3.64%, respectively) (Fig. 7a, b, c and d). Pyruvic acid is an improtant precursor of organic acids and plays a key role in primary metabolism. At the same time, it is also the linker of glycolysis pathway and TCA cycle [34]. Compared with the control conditions, the pyruvic acid content of PQ increased by 46.43%, while it decreased by 13.17% in PB (Fig. 7e). Hexokinase (HK), phosphofructokinase (PFK), and pyruvate kinase (PK) are the three most important irreversible enzymes in the glycolytic pathway. Under cold stress, HK, PFK and PK activities of PQ increased by 5.56, 60.43 and 126.92%, respectively, compared with the control conditions (Fig. 7f, g and h). However, in PB, the activities of PFK and PK were significantly lower than those in the control conditions, with the exception of HK activity (Fig. 7f, g and h).

**Fig. 8:**
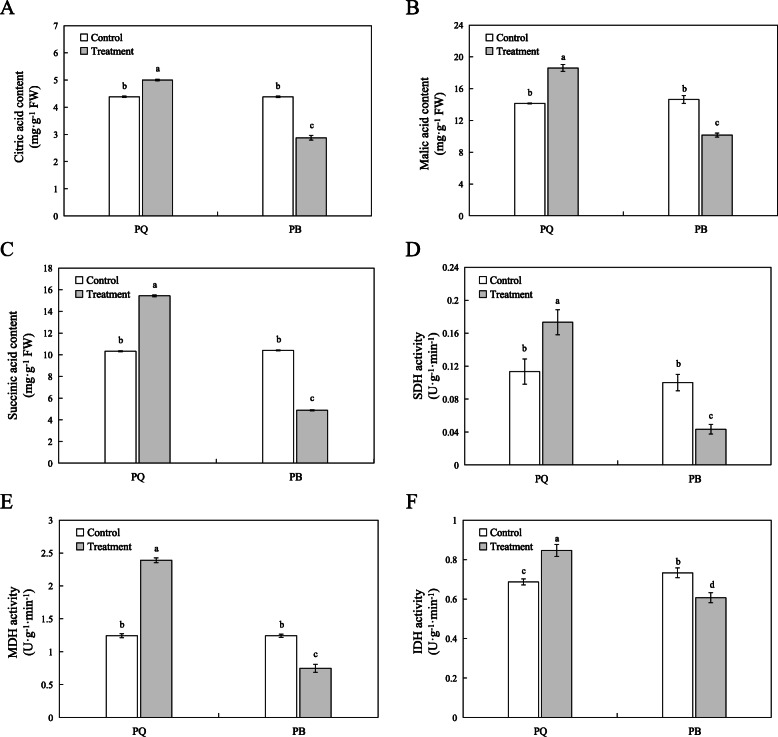
Effect of the citrate cycle (TCA cycle) products content and related enzyme activities of two *P.pratensis* cultivars under cold stress. The citric acid (**a**), malic acid (**b**), succinic acid content (**c**), SDH (**d**), MDH (**e**), IDH (**f**) activity were measured. All data are presented as means±SE from three independent experimental replicates. Different letters indicate significantly different at *p* < 0.05

Citric acid, malic acid and succinic acid are the main organic acids in the TCA cycle pathway. In this study, the cold stress significantly increased the level of citric acid, malic acid and succinic acid in PQ by 13.47, 33.50 and 49.37%, respectively, compared with the control conditions (Fig. 8a, b and c). However, the cold stress significantly reduced the level of these three organic acids in PB (Fig. 8a, b and c). Succinate dehydrogenase (SDH), malate dehydrogenase (MDH) and isocitrate dehydrogenase (IDH) are several key enzymes in the TCA cycle pathway. The results showed that the SDH, MDH and IDH activities of PQ were significantly improved compared with the control conditions (Fig. 8d, e and f). However, in PB, the activities of SDH, MDH and IDH were significantly lower than those in the control conditions (Fig. 8d, e and f).

### Verification of DEGs using quantitative qRT-PCR

**Fig. 9:**
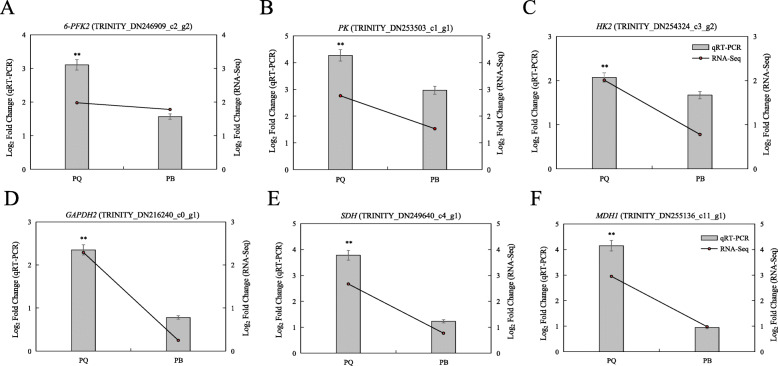
Expression patterns of selected genes involved in cold stress in two *P.pratensis* cultivars. Histograms were used to describe qRT-PCR, and line charts were used to describe RNA-Seq. All data are presented as means±SE from three independent experimental replicates. The asterisks denote significant differences (٭*p* < 0.05, ٭٭*p* < 0.01) as determined by Student’s *t-*tests

To validate the data obtained by RNA-seq, we selected 6 DEGs for qRT-PCR. These 6 genes, as the representative key genes in the glycolysis and TCA cycle, were involved in cold stress tolerance. Our results showed that the expression levels of these 6 genes in PQ was significantly higher than that in PB under cold stress (Fig. 9). This indicated that the high expression levels of these representative key genes were an important functional factor of the PQ adaptation to cold stress. Although there were differences in fold-changes, the expression pattern of selected genes analyzed by qRT-PCR was similar to that obtained by the RNA-Seq method, which also confirmed the reliability of the RNA-Seq results.

## Discussion

Low temperature is a common environmental stress factor for plants. Plants respond to cold stress by changing their morphology and physical and biochemical properties [35]. Molecular and genetic studies have shown that these changes may include the expression of numerous related genes [36]. RNA-seq has provided a new avenue for research on the responses of plants to cold stress. Therefore, using RNA-seq technology to understand the mechanisms of physiological responses and genes involved in cold stress signaling networks is a key to cold-tolerance breeding of plants. In this study, we used RNA-seq technology to analyze and compare the transcriptome of the cold-tolerance *P.pratensis* var. *anceps* cv. Qinghai (PQ) and the cold-sensitive *P.pratensis* cv. ‘Baron’ (PB) in response to cold stress to identify genes putatively involved in cold stress tolerance in PQ. The results are helpful for better understanding the molecular mechanism of PQ in response to cold stress and could be useful for cultivating *P.pratensis* varieties with tolerance to cold stress. Some characteristics of the cold response in PQ are discussed below.

Changes in carbohydrate content directly affect plant physiological activities, such as photosynthesis, respiration and metabolic processes [37]. A recent report showed that the carbohydrate metabolism pathway of loquat was the most sensitive under cold stress, and the DEGs obtained by transcriptome sequencing were mainly related to the glycolysis, pyruvate metabolism, starch and sucrose metabolism, pentose and glucuronate interconversions and fructose and mannose metabolism pathways [38]. These observations are consistent with our results. Our results also show that the carbohydrate metabolism in *P.pratensis* was the most sensitive to cold stress. In these two *P.pratensis* cultivars, many DEGs related to carbohydrate metabolism were maped to the “starch and sucrose metabolism”, “glycolysis” and “galactose metabolism” pathway, including 225 in PQ and 112 in PB (Fig. 4 and Additional file 6: Figure S3). Under cold stress, carbohydrates can act as osmotic protectants to stabilize cell membranes and remove ROS, thereby protecting plants from oxidative damage [39, 40]. Some studies have shown that the fructan content and fructosyltransferase gene transcription induction observed in cold-tolerance ‘Falster’ *Lolium perenne* were higher than those of cold-sensitive ‘Veyo’ *Lolium perenne* [24]. In addition, a large amounts of starch, sucrose, and hexose were accumulated in leaves of muskmelon under cold stress [41]. These studies showed that some carbohydrate contents in cold-tolerance genotype were higher than in cold-sensitive genotype. In our study, there was greater accumulation of total soluble sugars, starch, sucrose, fructose and glucose in cold-tolerance PQ than in cold-sensitive PB, which agrees with previous reports (Fig. 1f and Fig. 7a, b, c and d). In addition, we found that the O_2_^·-^ generation rate and H_2_O_2_ content of PQ were both lower than those of PB (Fig. 1g and h), which indicated that carbohydrate also played a role in stabilizing cell membranes and removing ROS. There are many kinds of carbohydrates in plants [42–44]. Different kinds of carbohydrates can participate in maintaining stability of cell membrane structure function [45]. However, there are also significant differences in the types of carbohydrates accumulated by different varieties of plants under cold stress. Previous studies have reported that, under cold stress, raffinose accumulates more in *Arabidopsis* [42], while sucrose and fructose are mainly in *Triticum aestivum* [43] and *Secale cereale* [44]. Our results show that the sucrose content of PQ and PB increased significantly under cold stress, and the increase of PQ was significantly higher than that of PB (Fig. 7b), which indicated that the accumulation of sucrose in the whole stress process mainly affected the cold tolerance of these two *P.pratensis* cultivars. Our study and others have shown that sucrose is one of the soluble carbohydrates, which has a potential role in plant adaptation to abiotic stress [46]. Previous studies showed that both glucose and ABA induced the expression of key regulatory genes of abiotic stress, such as *CBF3*, *COR15A* and *RD29A* [47, 48]. In addition, some jasmonate, abscisic acid and *COR* genes are controlled by soluble sugars [48]. Therefore, these results suggested that carbohydrates play a crucial role in the PQ response to cold stress.

The glycolysis pathway is not only one of the main pathways of plant respiration, but also an important pathway for regulating carbohydrate metabolism. Glucose and fructose are further metabolized by the glycolysis pathway, producing energy (ATP), reducing agent (NADH) and pyruvate acid [49]. Under many abiotic stresses, glycolysis is often involved in regulating the adaptability of plants to the environment [50]. Previous studies have reported that wheat and rice can regulate genes related to glycolysis pathway in response to Al stress [51, 52]. In the present study, we compared the KEGG pathway of PQ and PB, and found that both genotypes were enriched in the glycolysis pathway, but the DEGs enrichment in PQ was significantly higher than in PB (Fig. 5). Forty nine DEGs were identified from PQ to participate in the glycolysis pathway, and 40 DEGs encoding key enzymes related to glycolysis were significantly upregulated (Fig. 5b). In addition, we selected four key DEGs of the glycolysis pathway for qRT-PCR validation, and the results showed that the expression levels of these DEGs were increased under cold stress, but the increase in PQ was greater than that in PB. These result indicate that PQ can effectively regulate the expression level of key genes in glycolysis pathway to alleviate the inhibition caused by cold stress. The key enzymes in glycolysis pathway mainly include hexokinase (HK, EC 2.7.1.1), 6-phosphofructokinase (PFK, EC 2.7.1.11), glyceraldehyde 3-phosphate dehydrogenase (GAPDH, EC 1.2.1.12) and pyruvate kinase (PK, EC 2.7.1.40), etc. The glycolysis pathway is a complex metabolic process that is affected by related enzyme activities [53]. HK, PFK, and PK are the key rate-limiting enzymes in the glycolysis pathway. Previous studies showed that low levels of HK activity can inhibit plants senescence and regulate plants permeability to adapt to the environment [54, 55]. In this study, we found that the HK activity of PQ and PB increased significantly after cold stress, but the increase of PQ was less than that of PB (Fig. 7f), which indicated that HK with low activity could improve the cold tolerance of plants. Studies have found that reducing PK and PFK activity can inhibit glycolysis pathway and other pathways related to intermediate metabolism [55]. Our study found that cold stress reduced the activity of PFK and PK in PB, which would be harmful to plant respiratory metabolism (Fig. 7g and h). On the contrary, the activities of PFK and PK in PQ were significantly increased, which accelerated the glycolysis metabolism (Fig. 7g and h). Accordingly, the genes encoding these enzymes also increased their expression level. The increase of PFK activity can accelerate the transformation from fructose-6-phosphate to fructose-1,6-bisphosphate, and provide more oxaloacetic acid and pyruvic acid for TCA cycle [56]. Pyruvic acid is an important organic acid that connects the glycolysis pathway with the TCA cycle [34]. In the present study, the pyruvate acid content in PQ was significantly increased under cold stress, which provided more substances for the TCA cycle (Fig. 7e). Our RNA-seq data and in-depth analysis of glycolysis metabolism indicated that the glycolysis pathway plays an important role in the adaptation mechanism of PQ to cold stress response.

The TCA cycle is not only a bridge connecting carbohydrate, amino acid, lipid and protein metabolism, but also an engine to generate energy and reducing power to drive metabolism [57]. There are eight enzymes in the TCA cycle, which are citrate synthase (CSY, EC 2.3.3.1), aconitase (ACO, EC 4.2.1.3), isocitrate dehydrogenase (IDH, EC 1.1.1.41), α-ketoglutarate dehydrogenase (αKGDH, EC 1.2.4.2), succinyl-CoA synthetase (SCoAL, EC 6.2.1.4), succinate dehydrogenase (SDH, EC 1.3.5.1), fumarase (FUM, EC 4.2.1.2), and malate dehydrogenase (MDH, EC 1.1.1.37). Among the TCA mutants of tomato, FUM, MDH and αKGDH play a key role in regulating the TCA cycle [58]. Under abiotic stress, the TCA cycle is one of the important protection systems for plants [59]. Recently, Wu et al. [60] studied the transcriptome and metabolome of *Dendrobium officinale* under cold stress, which showed that the accumulation of several important intermediate products in TCA cycle, such as citric acid, succinic acid and fumaric acid, resulted in the increase of activities of isocitrate lyase (ICL), ACO and SDH. Our RNA-seq data showed that 16 DEGs were enriched in the TCA cycle pathway in PQ, while only 3 DEGs were enriched in PB (Fig. 6b and c). The 16 DEGs enriched in PQ were mainly involved in encoding key enzymes of the TCA cycle pathway, such as MDH and SDH (Fig. 6a). Our study has found that cold stress significantly reduced the activities of MDH, SDH and IDH, the key enzymes of TCA cycle in PB (Fig. 8d, e and f). As a result, the TCA cycle was inhibited, the production of organic acids was reduced, and ultimately the metabolic rate was reduced, which inhibited the mitochondrial electron transport chain [61] and affected the normal physiological metabolism of PB (Fig. 8a, b and c). However, the activities of MDH, SDH and IDH in PQ were significantly increased under cold stress, resulting in the increase of citric acid, malic acid and succinic acid (Fig. 8). It is worth noting that MDH activity increased the most. These help the TCA cycle to function normally and provide more organic substances for other metabolic pathways. The TCA cycle contributes to the energy metabolism of cells, and the expression of genes related to energy metabolism is very important for the growth and development of plants under stress [62]. Generally, photosynthesis is the main process of energy source of plant cells. Under stress, photosynthesis is blocked, glycolysis and TCA cycle will provide energy for cells [59]. These results indicated that PQ can respond to cold stress by actively regulating glycolysis and TCA cycle pathway related genes and enzyme activities. Therefore, revealing the molecular functions of glycolysis and TCA cycle genes can provide a deeper understanding of plant development.

## Conclusions

**Fig. 10:**
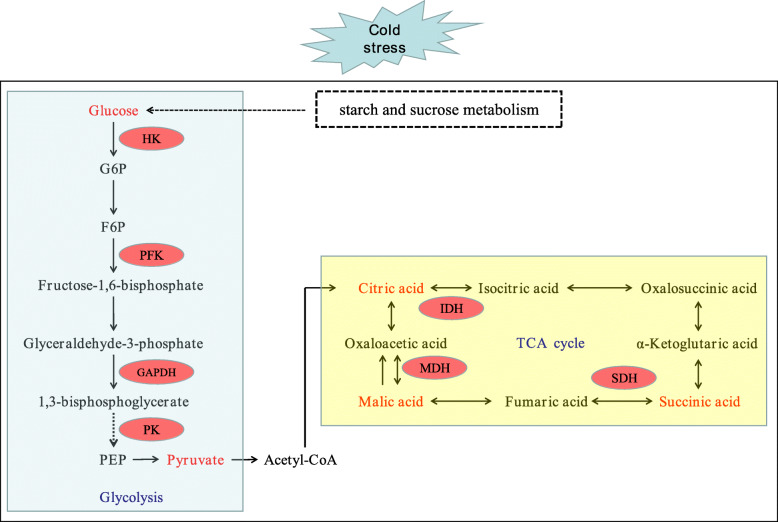
Schematic presentation of the potential mechanism of *P.pratensis* var. *anceps* cv. Qinghai tolerance to cold stress. Red indicates up-regulation

As one of the variant of *P.pratensis*, *P.pratensis* var. *anceps* cv. Qinghai is more cold tolerance than the commercially cultivated varieties of *P.pratensis*. In this study, we analyzed the physiological regulation and transcriptional changes of *P.pratensis* var. *anceps* cv. Qinghai and the cold-sensitive *P.pratensis* cv. ‘Baron’ under cold stress. We identified 5996 and 3285 DEGs between the treatment vs control comparison of *P.pratensis* var. *anceps* cv. Qinghai and *P.pratensis* cv. ‘Baron’, respectively, with 5612 DEGs specific to *P.pratensis* var. *anceps* cv. Qinghai. Genes related to glycolysis and citrate cycle (TCA cycle), such as *HK*, *PFK*, *GAPDH*, *PK*, *SDH* and *MDH*, were found to be involved in the tolerance to cold stresses in *P.pratensis* var. *anceps* cv. Qinghai. The further study showed that *P.pratensis* var. *anceps* cv. Qinghai could improve the expression level of related genes, regulate the activities of related enzymes in glycolysis and TCA cycle, which significantly increased the production of organic acids and pyruvate acid, and provided more material and energy for defense against cold stress, and thus improved the cold tolerance of *P.pratensis* var. *anceps* cv. Qinghai. Accordingly, we established a hypothesis model of glycolysis and TCA cycle in response to cold stress (Fig. 10). Our study not noly provides important insights into the molecular mechanisms of *P.pratensis* var. *anceps* cv. Qinghai responds to cold stress, but also systematically reveals the changes of key genes and products of glycolysis and TCA cycle in response to cold stress, which is conductive to the breeding of cold-tolerance *P.pratensis* genotype.

## Methods

### Plant materials and cold treatment

Seeds of *Poa pratensis* var. *anceps* cv. Qinghai (PQ) were obtained from Qinghai Academy of Animal Science and Veterinary Medicine. Qinpin Zhou (Qinghai Academy of Animal Science and Veterinary Medicine) undertook the formal identification of PQ and obtained legal permission (samples NO.278) on April 20, 2005. However, there is no publicly available herbarium to deposite the voucher specimen of this material at present. Seeds of *Poa pratensis* cv. ‘Baron’ (PB) were obtained from the Beijing Clover Grass Technology Development Center (Beijing, China), which is a commercial variety of *Poa pratensis*. The seeds of these two genotypes were grown in the artificial climate chamber of Gansu Agricultural University, Lanzhou Gansu, China, under the control conditions: (25 ± 1)/(20 ± 1) °C during a 16/8 h light/dark cycle, light density of 20–40 μm·s^− 1^·m^− 2^, and relative humidity of 50–60%. Two-months-old seedlings were transferred to the low temperature artificial climate chamber at 0 °C, and the seedlings kept at 25 ± 1 °C were treated as control conditions. After 72 h, the whole leaf plates from three independent cold-treated or control branches were collected. Phenotypic observation was performed 24 h after recovery at room temperature. All samples were frozen in liquid nitrogen and stored at − 80 °C for RNA extraction and physiological analysis. Three independent experimental replicates were performed, each consisting of three independent biological replicates.

### Analysis of physiological and *CBF*s expression levels

The relative electrolyte conductivity (REC) was determined according to Lutts et al. [63]. The malondialdehyde (MDA) content was measured by the thiobarbituric acid method [64]. The free proline (Pro) content was determined by the sulfosalicylic acid-acid ninhydrin method [65]. The soluble sugar (SS) content was measured by the anthrone method [66]. The superoxide radical (O_2_^·-^) generation rate was determined by hydroxylamine oxidation [67]. Hydrogen peroxide (H_2_O_2_) was measured according to Willekens et al. [68]. The superoxide dismutase (SOD, EC 1.15.1.11) activity was assayed according to Giannopolitis and Ries [69]. The peroxidase (POD, EC 1.11.1.7) activity was measured according to Chance and Maehly [70]. The activities of catalase (CAT, EC 1.11.1.6) and ascorbate peroxidase (APX, EC 1.11.1.11) were determined according to Xu et al. [71]. The specific primers of C-repeat binding factors (*CBF*s) were designed according to Zhang et al. [29]. In qRT-PCR analysis of *CBF*s, *Actin* gene was used as an internal control [72], the expression data were normalized to the control conditions (set at 1), using the comparative threshold cycle method.

### RNA extraction and quality control

Total RNA was extracted from the leaves using TRIzol® Reagent (Tiangen Biotech, Beijing, China) according to the manufacturer’s instructions. RNA degradation and contamination were monitored using 1% agarose electrophoresis. RNA purity was evaluated using a Nanodrop Spectrophotometer (SpectraMax® QuickDropTM, Molecular Devices, Shanghai, China). RNA integrity was evaluated using an Agilent 2100 Bioanalyzer (Agilent Technologies, CA, USA), and only RNA samples with RNA integrity numbers (RINs) ranging from 8 to 10 were further analyzed.

### Illumina cDNA library construction and sequencing

After the RNA samples were tested, the mRNA was enriched using magnetic beads with Oligo (dT). Subsequently, fragmentation buffer was used to fragment the mRNA into short fragments. Based on the short mRNA fragments, random hexamer primers were used to synthesize the first-strand cDNA, and the double-stranded cDNA was subsequently synthesized using DNA polymerase I and RNase H. The double-stranded cDNA was purified using AMPure XP beads and then end repaired, and adaptor ligation was carried out after the addition of the ‘A’ tail. Finally, PCR was performed, and the PCR products were purified using AMPure XP beads to obtain the final cDNA library. The cDNA library was constructed and sequenced using the Illumina HiSeq2500 platform by Major Biomedical Technology Co., Ltd. (Shanghai, China).

### Transcriptome assembly and gene functional annotation

The raw data obtained by Illumina sequencing were analyzed by CASAVA (v 1.70) base recognition and then converted into the original sequences to obtain the raw reads. Clean reads were obtained by removing reads containing adaptors, reads containing more than 5% ambiguous bases (“N”) and low-quality reads (> 50% of the bases with a Q-value ≤5). Clean reads were de novo assembled by Trinity (v 2.90) (http://trinityrnaseq.github.io). The longest transcript was selected from the spliced transcript sequence as the “unigene”, and all transcripts and unigenes were counted and used for subsequent bioinformatic analyses.

All assembled unigenes were aligned and annotated in the Non-Redundancy Protein (NR) (http://www.ncbi.nlm.nih.gov/), Swiss-Prot (http://www.expasy.ch/sprot/), Pfam (http://pfam.xfam.org/), Cluster of Orthologous Groups of proteins (COG) (http://www.ncbi.nlm.nih.gov/COG), Gene Ontology (GO) (http://www.geneontology.org), and Kyoto Encyclopedia of Genes and Genomes (KEGG) (http://www.genome.jp/kegg/) databases using the BLASTx tool (v 2.2.28+) with an E-value ≤10^− 5^ threshold. The best BLAST hit was used to determine the sequence direction of the unigenes.

### In-depth analysis of differential gene expression

The transcriptome sequences obtained by Trinity (v 2.90) splicing were used as a reference sequence, and the clean reads of each sample were mapped to the reference sequence by RSEM (v 1.3.1) (http://deweylab.biostat.wisc.edu/rsem/). The expression level of each unigene was analyzed by calculating the transcripts per kilobase of exon model per million mapped reads (TPM). DESeq (v 1.20.0) software was used to identify DEGs in pairwise comparisons. DEGs between treated and control samples were identified with a false discovery rate (FDR) ≤ 0.05. Genes with a *P*-value (Padjust) < 0.01 and an absolute value of log_2_fold-change ≥1 found by DESeq were considered differentially expressed. GO enrichment analysis of DEGs was implemented by the GOseq (v 1.24.0) R package in which gene length bias was corrected. KEGG pathway enrichment analysis of genes or transcripts in gene sets was performed using the process developed by Major Biomedical Technology Co., Ltd. The calculation principle was the same as that in the GO function enrichment analysis. When the corrected P-value (Padjust) < 0.05, there was considered to be a significant enrichment in that KEGG pathway.

### Measurements of carbohydrate, glycolysis and TCA cycle products content and related enzyme activities

The sucrose, fructose and glucose content was determined according to Gomes et al. [73] with some modification. Specifically, 1 g fresh leaves were ground with liquid nitrogen and stored at − 20 °C until lyophylization. 0.5 g of lyophilized material was homogenized in 5 mL of deionized water. The mixture was centrifuged and filtered to obtain the supernatant. 0.2 mL supernatant was purified and analyzed by high-performance liquid chromatography (HPLC) as described by Gomes et al. [73]. The starch content was determined by the enzymatic method of Gomes et al. [73]. The pyruvic acid content was determined by the Schwimmer and Weston method [74], modified by Anthon [75]. The hexokinase (HK) activity was assayed according to Schaffer and Petreikov [76]. The phosphofructokinase (PFK) activity was measured according to Mustroph et al. [77]. The pyruvate kinase (PK) was determined according to Schweizer and Erismann [78].

Citric acid, malic acid and succinic acid were determined according to Wang et al. [79]. Specifically, 1 g fresh leaves were ground with liquid nitrogen and added with 5 mL ethanol. The mixture was centrifuged and filtered through a 0.45 μm membrane to obtain the supernatant for purification and analysis by HPLC. At 25 °C, the separation was performed on a reversed-phase C-18 column (250 mm × 4.5 mm, 5 μm, Solarbio, Beijing, China). The mobile phase was 5:95 (v/v) methanol: water and the flow rate was 0.8 mL·min^− 1^. Organic acids were detected at 210 nm using potassium dihydrogen phosphate solution (pH 2.7) as mobile phase. The corresponding peaks of organic acids (citric acid, malic acid and succinic acid) were determined by comparing their retention times with known standards. The succinate dehydrogenase (SDH) activity was measured according to Green et al. [80]. The malate dehydrogenase (MDH) activity was determined using the method of Dannel et al. [81]. The isocitrate dehydrogenase (IDH) activity was measured according to Bergmeyer et al. [82].

### Quantitative real-time PCR (qRT-PCR) validation

To verify the reliability and accuracy of the RNA-seq data, six genes were selected for validation by qRT-PCR. Total RNA was extracted from the control and cold groups of the two genotypes using TRIzol® Reagent (Tiangen Biotech, Beijing, China) according to the manufacturerʼs instructions. cDNA was synthesized using the PrimeScript™ 1 st Strand cDNA Synthesis Kit (Solarbio, Beijing, China) according to the manufacturerʼs instructions. The cDNA was diluted and amplified using a Light Cycl®96 Real-Time PCR system (Roche Life Science, Shanghai, China) and SYBR® Premix Ex TaqTM II kit (Takara Biomedical Technology, Dalian, China) according to the manufacturerʼs instructions. The reaction conditions were 94 °C for 5 min, followed by 40 cycles of 95 °C for 15 s and 60 °C for 1 min. The relative level of gene expression was calculated using the 2^-ΔΔct^ formula [83]. The *P.pratensis* putative *Actin* gene was used as an endogenous control [72]. The specific primers (Additional file 7: Table S4) were designed with Primer software (v 6.24, Primer, Quebec City, Canada). qRT-PCR analysis included three independent technical repeats with three biological replicates.

## Supplementary information

**Additional file 1: Table S1.** Overview of the RNA sequencing data.

**Additional file 2: Table S2.** Summary statistics of the common vetch transcriptome assemblies.

**Additional file 3: Table S3.** Functional annotations of unigenes in the NR, Swiss-Prot, PFAM, COG, GO and KEGG databases

**Additional file 4: Figure S1.** Functional annotation of assembled transcriptome. (**A**) Species distribution of the top BLAST hits; (**B**) Map of GO functional categories; (**C**) Map of COG function classifcations.

**Additional file 5: Figure S2.** GO annotation of the DEGs between PQ and PB under cold stress.

**Additional file 6: Figure S3.** KEGG pathway classification of PQ (**A**) and PB (**B**).

**Additional file 7: Table S4**. List of primers used for qRT-PCR.

## Data Availability

Raw Illumina sequence data were deposited in the National Center for Biotechnology Information (NCBI) and be accessed in the sequence read archive (SRA) database (https://www.ncbi.nlm.nih.gov/sra). The accession number is PRJNA598031 (https://www.ncbi.nlm.nih.gov/bioproject/PRJNA598031), which includes 12 accession items (SRR10800767-SRR10800778). All data generated or analysed during this study are included in this published article and the it supplementary information files.

## Abbreviations

PQ: *P.pratensis* var. *anceps* cv. Qinghai
PB: *P.pratensis* cv. ‘Baron’
RNA-seq: RNA sequencing
DEGs: Differentially expressed genes
KEGG: Kyoto Encyclopedia of Genes and Genomes
ROS: Reactive oxygen species
ABA: Abscisic acid
DREB1: Dehydration-responsive element-binding protein 1 s
CBFs: C-repeat binding factors
MAPK: Mitogen-activated protein kinase
COR: Cold-responsive
DRE/CRT: Dehydration-responsive-element/C-repeat
REC: Relative electrolyte conductivity
MDA: Malondialdehyde
Pro: Free proline
SS: Soluble sugar
H_2_O_2_: Hydrogen peroxide
SOD: Superoxide dismutase
POD: Peroxidase
CAT: Catalase
APX: Ascorbate peroxidase
RINs: RNA integrity numbers
NR: Non-Redundancy Protein
COG: Cluster of Orthologous Groups of proteins
GO: Gene Ontology
FPKM: The fragments per kb per million reads
FDR: False discovery rate
HPLC: High-performance liquid chromatography
HK: Hexokinase
PFK: Phosphofructokinase
PK: Pyruvate kinase
SDH: Succinate dehydrogenase
MDH: Malate dehydrogenas
IDH: Isocitrate dehydrogenase
CSY: Citrate synthase
ACO: Aconitase
αKGDH: α-ketoglutarate dehydrogenase
SCoAL: Succinyl-CoA synthetase
FUM: Fumarase
ICL: Isocitrate lyase
qRT-PCR: Quantitative Real-Time PCR
