# Supplementary material for: Physiological and transcriptome analysis of Poa pratensis var. anceps cv. Qinghai in response to cold stress
Source: BMC Plant Biol. 2020 Jul 31;20:362. doi: 10.1186/s12870-020-02559-1 (PMC7393922; doi:10.1186/s12870-020-02559-1)
Supplement: Supplementary file 4 — Additional file 4: Figure S1. Functional annotation of assembled transcriptome. (A) Species distribution of the top BLAST hits; (B) Map of GO functional categories; (C) Map of COG function classifcations. [file 12870_2020_2559_MOESM4_ESM.doc]

| A  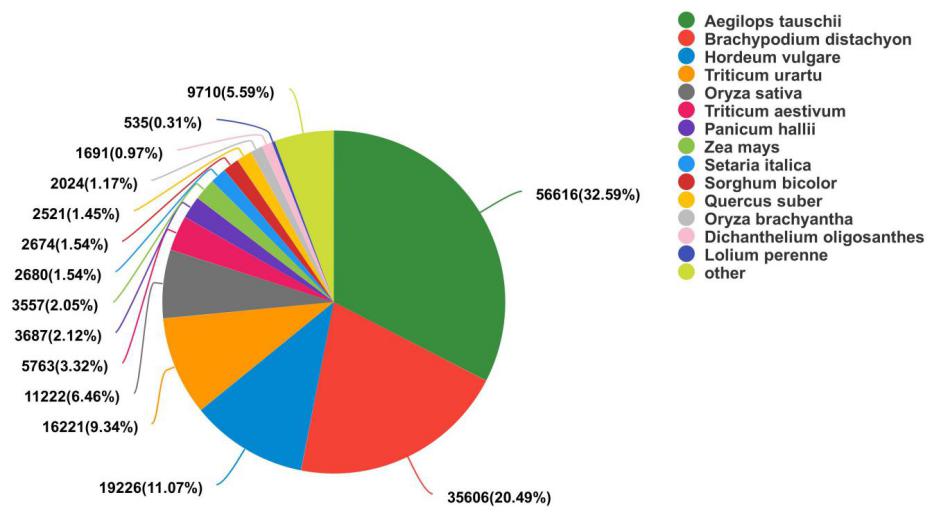 |
| --- |
| B  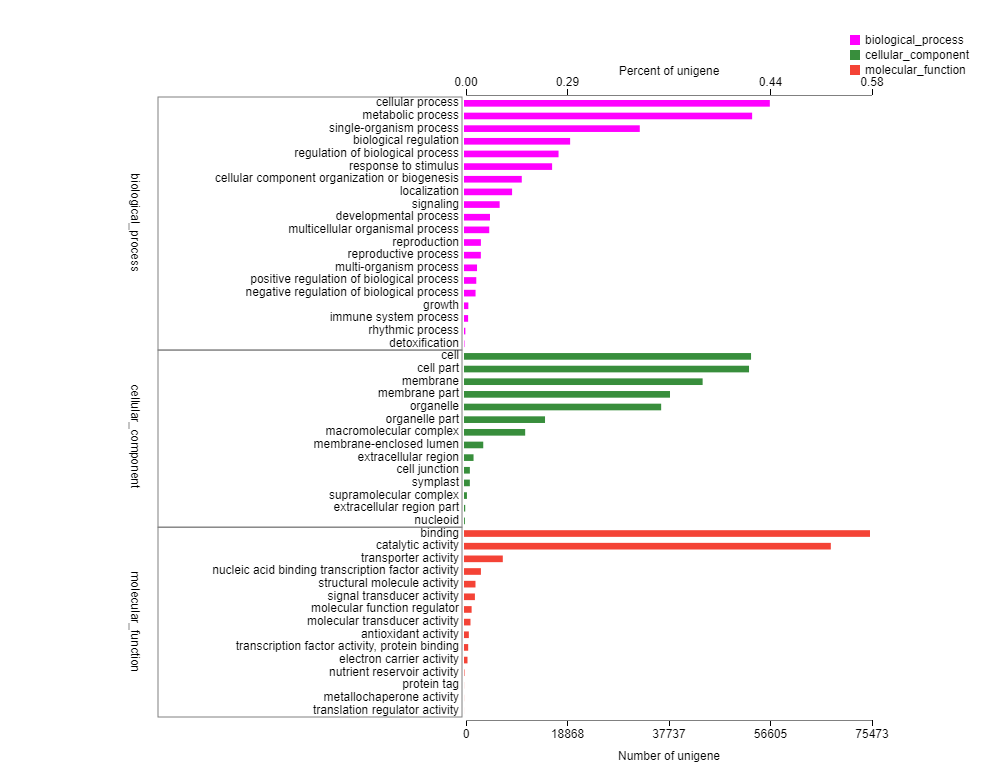 |
| C  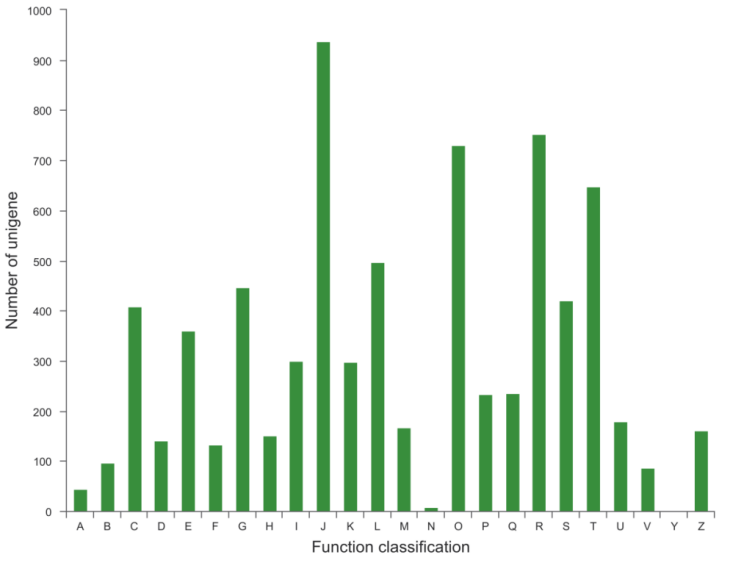 |

**Fig. S1** Functional annotation of assembled transcriptome. (**A**) Species distribution of the top BLAST hits; (**B**) Map of GO functional categories; (**C**) Map of COG function classifcations. A: RNA processing and modification; B: Chromatin structure and dynamics; C: Energy production and conversion; D: Cell cycle control, cell division, chromosome partitioning; E: Amino acid transport and metabolism; F: Nucleotide transport and metabolism; G: Carbohydrate transport and metabolism; H: Coenzyme transport and metabolism; I: Lipid transport and metabolism; J: Translation, ribosomal structure and biogenesis; K: Transcription; L: Replication, recombination and repair; M: Cell wall/membrane/envelope biogenesis; N: Cell motility; O: Posttranslational modification, protein turnover, chaperones; P: Inorganic ion transport and metabolism; Q: Secondary metabolites biosynthesis, transport and catabolism; R: General function prediction only; S: Function unknown; T: Signal transduction mechanisms; U: Intracellular trafficking, secretion, and vesicular transport; V: Defense mechanisms; Y: Nuclear structure; Z: Cytoskeleton
