# Supplementary material for: Physiological and transcriptome analysis of Poa pratensis var. anceps cv. Qinghai in response to cold stress
Source: BMC Plant Biol. 2020 Jul 31;20:362. doi: 10.1186/s12870-020-02559-1 (PMC7393922; doi:10.1186/s12870-020-02559-1)
Supplement: Supplementary file 5 — Additional file 5: Figure S2. GO annotation of the DEGs between PQ and PB under cold stress. [file 12870_2020_2559_MOESM5_ESM.doc]

|  |
| --- |

**Fig. S2** GO annotation of the DEGs between PQ and PB under cold stress
