# Supplementary figures and images for: Physiological and transcriptome analysis of Poa pratensis var. anceps cv. Qinghai in response to cold stress
Source: BMC Plant Biol. 2020 Jul 31;20:362. doi: 10.1186/s12870-020-02559-1 (PMC7393922; doi:10.1186/s12870-020-02559-1)

| A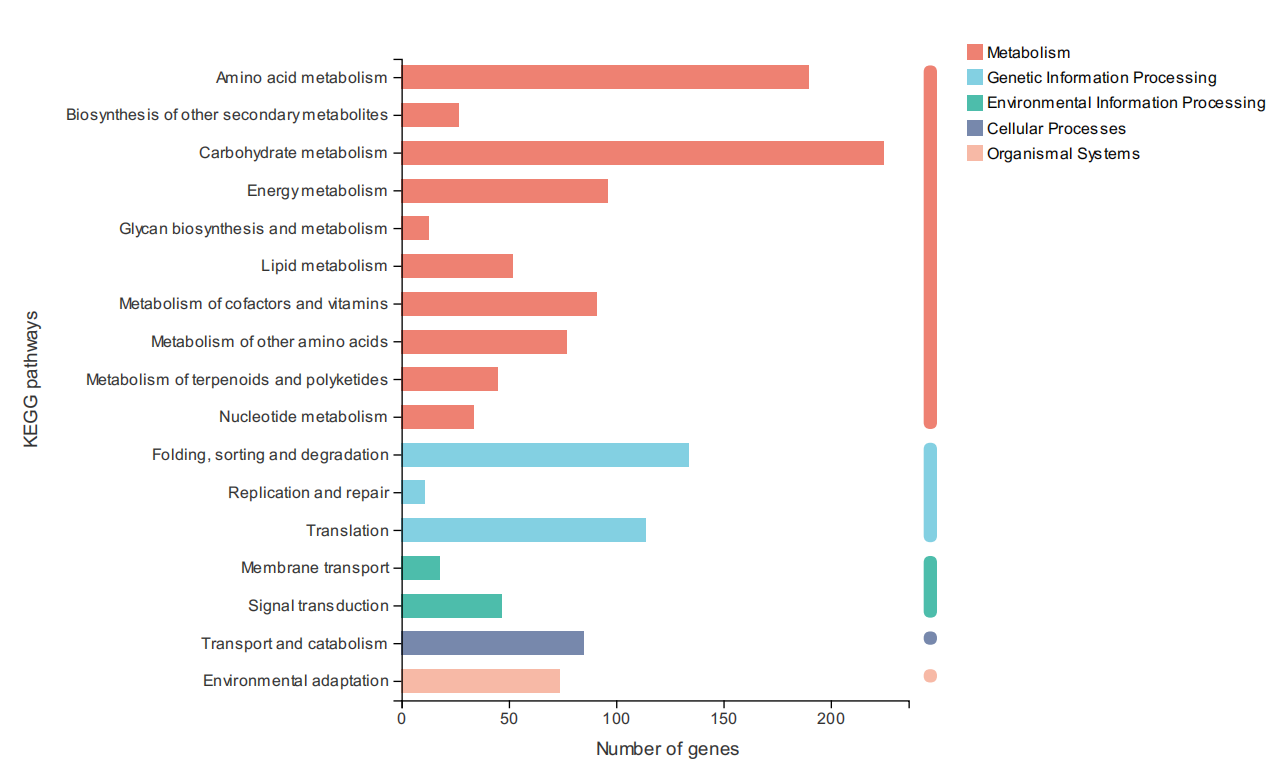 |
| --- |
| B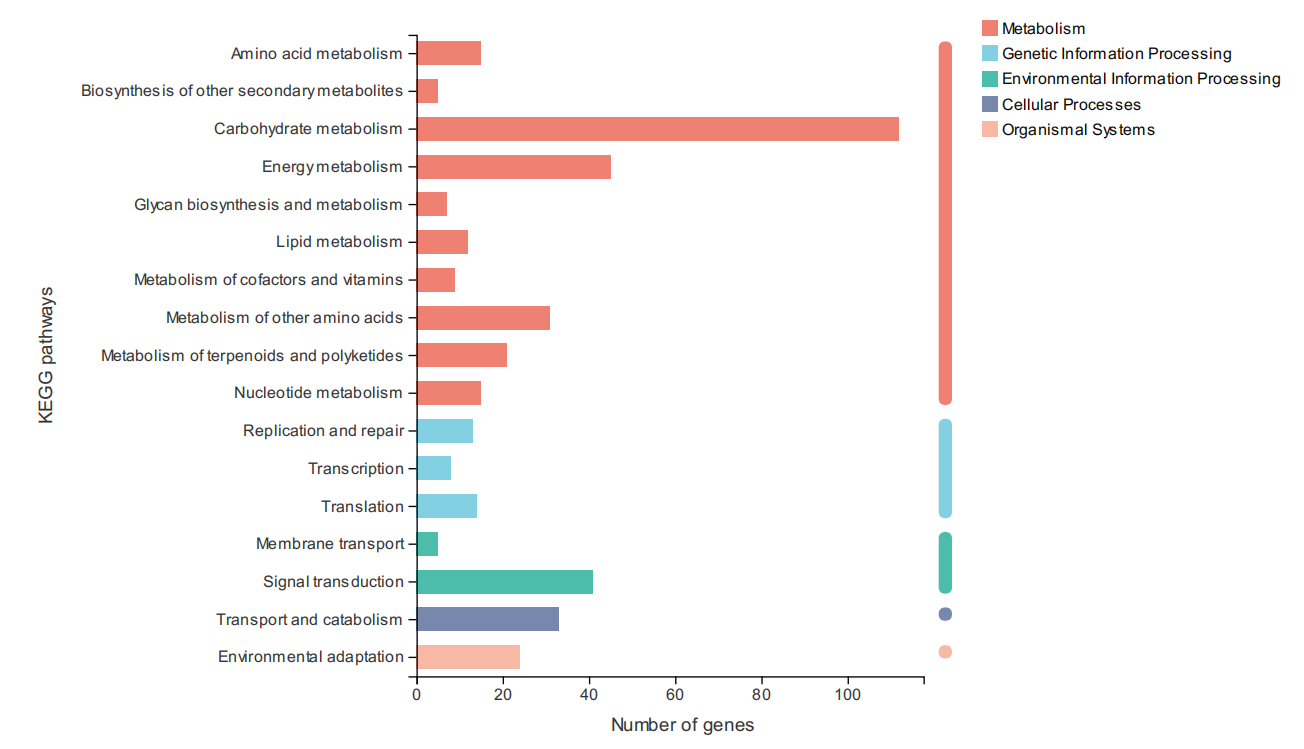 |

**Fig. S3** KEGG pathway classification of PQ (**A**) and PB (**B**).

Supplement: Supplementary file 6 — Additional file 6: Figure S3. KEGG pathway classification of PQ (A) and PB (B). [file 12870_2020_2559_MOESM6_ESM.doc]
